# Variation in Terpenoid and Flavonoid Content in Different Samples of *Salvia semiatrata* Collected from Oaxaca, Mexico, and Its Effects on Antinociceptive Activity

**DOI:** 10.3390/metabo13070866

**Published:** 2023-07-20

**Authors:** Nancy Ortiz-Mendoza, Rubén San Miguel-Chávez, Martha Juana Martínez-Gordillo, Francisco Alberto Basurto-Peña, Mariana Palma-Tenango, Eva Aguirre-Hernández

**Affiliations:** 1 Laboratorio de Productos Naturales, Departamento de Ecología y Recursos Naturales, Facultad de Ciencias, Universidad Nacional Autónoma de México, Ciudad de México 04510, Mexico; nancy_om@ciencias.unam.mx; 2 Posgrado en Ciencias Biológicas, Unidad de Posgrado, Edificio D, 1° Piso, Circuito de Posgrados, Ciudad Universitaria Coyoacán, Ciudad de México 04510, Mexico; 3 Posgrado en Botánica, Colegio de Postgraduados, Campus Montecillo, Texcoco Estado de México 56230, Mexico; sanmi@colpos.mx; 4 Departamento de Biología Comparada, Herbario de la Facultad de Ciencias, Universidad Nacional Autónoma de México, Ciudad de México 04510, Mexico; mjmg@ciencias.unam.mx; 5 Jardín Botánico, Instituto de Biología, Universidad Nacional Autónoma de México, Ciudad de México 04510, Mexico; abasurto@ib.unam.mx; 6 Facultad de Ciencias, Universidad Nacional Autónoma de México, Ciudad de México 04510, Mexico; marianapt@ciencias.unam.mx

**Keywords:** Lamiaceae, terpenoids, flavonoids, neo-clerodane, variation secondary metabolism, antinociceptive effect

## Abstract

*Salvia semiatrata* Zucc. (Lamiaceae) is endemic to Oaxaca, Mexico, and is known for its analgesic properties. Terpenoids and phenolic compounds with antinociceptive potential have been characterised from this species. The aim of this research was to determine the variation in terpenoids and flavonoids in ethyl acetate extracts of *S. semiatrata* collected from ten different localities, as well as to evaluate the antinociceptive effect between plants with higher and lower contents of these secondary metabolites. Quantification of *S. semiatrata* compounds was performed via HPLC-DAD, whereas in vivo evaluation of the antinociceptive effect was performed via formalin test. The results showed that the most abundant groups of metabolites are oleanolic acid (89.60–59.20 µg/mg), quercetin (34.81–16.28 µg/mg), catechin (11.30–9.30 µg/mg), and 7-keto-neoclerodan-3,13-dien-18,19:15,16-diolide (7-keto) (8.01–4.76 µg/mg). Principal component and canonical correspondence analysis showed that the most contrasting localities in terms of compound content and climatic variables are Miahuatlán and Santiago Huauclilla. The differences in metabolite content between the two locations did not affect the antinociceptive effects evaluated at a dose of 300 mg/kg, p.o. In conclusion, the results indicate that *S. semiatrata* is effective in relieving pain, regardless of the site of collection, reinforcing its traditional use as analgesic.

## 1. Introduction

*Salvia* is the genus of the Lamiaceae family with the highest species diversity in Mexico, being represented by approximately 306 species, which are widely distributed in temperate forests, particularly coniferous and oak forests, although there are also sections that prefer arid areas [1,2]. Several species of *Salvia* have been reported to have a wide range of uses in Traditional Mexican Medicine, including alleviating respiratory diseases, such as bronchitis and coughs, and curing skin conditions, such as pimples, rashes, and measles. Likewise, they are used in the treatment of women’s health issues, such as menstrual cramps, vaginal bleeding, and coldness in the womb; as a tranquiliser; in cleansing rituals, and for treating fright, evil eye, headaches, and earaches. However, salvias are most often used against digestive disorders, including stomach pain, colic pain, empacho, dysentery, and diarrhoea [3,4]. Pharmacological studies on Mexican salvias have documented their antioxidant, antidiabetic, antimicrobial, antinociceptive, anti-inflammatory, and cytotoxic properties, highlighting the potent analgesic and anti-inflammatory effects of extracts and compounds that are terpenic and phenolic in nature [4,5]. The antinociceptive effects of organic and aqueous extracts, as well as isolated compounds, have been demonstrated in murine models. Examples are the neo-clerodane diterpene glycoside (amarisolide A) and the flavonoid pedalitin isolated from *S. amarissima* (syn. *S. circinata*) [6]. Similar effects were observed with organic and aqueous extracts of *S. purpurea* [7]. Analgesic effects of organic extracts and the neo-clerodane diterpene 7-keto-neoclerodan-3,13-dien-18,19:15,16-diolide (7-keto) isolated from the ethyl acetate extract of *S. semiatrata* have also been demonstrated, with this extract showing the greatest antinociceptive effect in writhing and formalin tests [8]. In recent phytochemical investigations carried out on *S. amarissima*, *S. involucrata*, *S. purpurea*, and *S. semiatrata*, the flavonoids apigenin, kaempferol, catechin, phloretin, phlorizin, galangin, myricetin, naringenin, quercetin, and rutin, as well as terpenoids, such as oleanolic and ursolic acids, α-amyrin, β-sitosterol, carsonol, and stigmasterol, have been identified through HPLC [6,7,8,9]. Most of the above compounds have been evaluated for their antinociceptive and anti-inflammatory properties, with good results identified via murine tests [5,10,11,12,13,14,15,16,17,18,19]. In the same way, Mexican salvias are a rich source of terpenoids, especially clerodane diterpenes (amarisolide A, 7-keto, salvinorin A, tilifodiolide, among others).

*Salvia semiatrata* Zucc. is an endemic subshrub found in the state of Oaxaca, Mexico. It is distributed in temperate forests and arid zones. Regionally, it is used as a healing, anti-inflammatory, earache, stomachache and nervous disorder treatment [4]. This sage has biological activities that have cytotoxic, antifungal, antibacterial, anxiolytic, and antinociceptive effects, and these beneficial effects result from the synthesis of both terpenoids and phenolic compounds [4]. However, it is known that the production of these metabolites varies quantitatively between plant organs, phenological stages, geographical origins, and the biotic and environmental contexts in which they develop. It has been observed that their synthesis is related to protection against abiotic stressors, such as high temperatures, drought, ultraviolet radiation, among others [20]. Despite the widespread interest in this topic, very little is known about the variation in compounds in species in response to these stressors and whether there is an influence on their biological effects. So far, there is no available information on the variability in the contents of bioactive compounds in the aerial parts of *S. semiatrata* and their impact on antinociceptive activity, which depends on the environment in which it develops. The aim of this research was to evaluate the quantitative variation in terpenoids and flavonoids, as well as the antinociceptive effect among wild populations of *S. semiatrata* collected at different sites in the state of Oaxaca.

## 2. Materials and Methods 

### 2.1. Drugs and Reagents

Diclofenac (DCF) and 37% Formalin were purchased from Merck (Mexico City, Mexico). Tween 80 and saline solution (SS) were purchased from Sigma (St. Louis, MO, USA). Ethyl acetate was purchased from Tecsiquim, S.A. de C.V. (Mexico City, Mexico). Terpenoid and flavonoid reference standards (99% purity) were purchased from Sigma Aldrich (St. Louis, MO, USA), and the neo-clerodane diterpene 7-keto-neoclerodan-3,13-dien-18,19:15,16-diolide was isolated and purified as a crystalline form by Ortiz-Mendoza et al., 2020. Crystallographic data were deposited at the Cambridge Crystallographic Data Centre (CCDC) using the number 1953351 [8].

### 2.2. Site Descriptions and Plant Material

**Figure 1:**
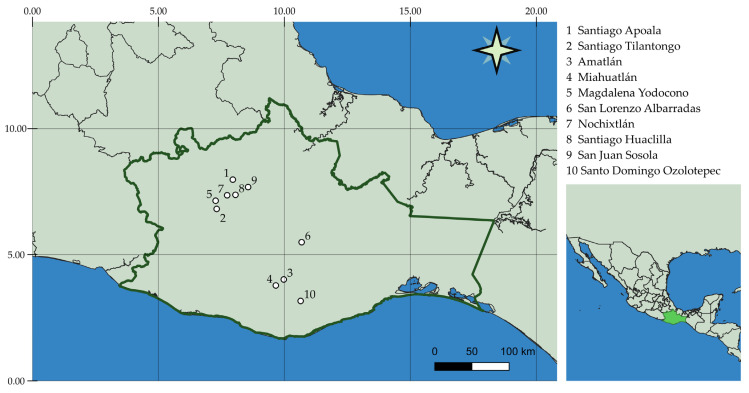
Locations of *S. semiatrata* populations in Oaxaca, Mexico, that were sampled for this study. Maps were generated using QGIS 3.28.1-Firenze.

*S. semiatrata* aerial parts were collected in June and November 2019 at ten different locations in Oaxaca, Mexico, which were based in the following municipalities: Amatlán (AMA), San Lorenzo Albarradas (ALB), San Juan Sosola (SOS), Santiago Huauclilla (HUA), Miahuatlán (MIA), Nochixtlán (NOX), Santo Domingo Ozolotepec (OZO), Magdalena Yodocono (YOD), Santiago Tilantongo (TIL) and Santiago Apoala (APO) (Figure 1 and Table 1). The plant material was identified by Ph.D. Martha J. Martínez Gordillo. Voucher specimens of these samples were deposited at the FCME herbarium of the Faculty of Sciences, the National Autonomous University of Mexico (UNAM). 

### 2.3. Preparations of the Extracts

Ethyl acetate (EtOAc) extracts sourced from the aerial parts of *S. semiatrata* were obtained via a maceration process that used 70 g of dry ground plant material. The powder was kept in a container immersed in solvent for 48 h at room temperature (22 °C). The plant material was filtered, and the liquid part was concentrated at a high vacuum level using a rotary evaporator (DLAB, SCI100-Pro, Shunyi, Beijing, China), leading to the production of EtOAc extract from *S. semiatrata.*

### 2.4. Analysis of Phenols and Terpenoids via High-Performance Liquid Chromatography (HPLC)

The extract was prepared at a concentration of 50 mg/mL of ethyl acetate of HPLC grade. Impurities were removed using nylon membrane acrodiscs with pores of 0.45 µm in size, and 20 µL of each of the extracts was injected. The extract of each sample was injected in triplicate. Extracts were analysed using a Hewlett Packard 1100 series HPLC with an autosampler (Agilent Technologies, 1200 Series Mod. G1329A, Santa Clara, CA, USA) and a diode array detector (Agilent Technologies, 1100 Series Mod., Santa Clara, CA, USA). For flavonoids, analyses were performed using a Hewlett Packard Hypersil ODS column (125 × 40 mm) with a gradient of (A) H_2_O at pH 2.5 using trifluoroacetic acid (TFA) and (B) acetonitrile (ACN), as well as the following parameters: flow rate, 1 mL/min; temperature, 30 °C; injection volumem 20 µL; λ1, 254 nm, λ2, 280 nm, and λ3, 330 nm; and analysis time, 25 min. The reference standards used were quercetin, catechin, and naringenin. For terpenoids, a zorbax Eclipse XDB-C8 column (125 × 4.0 mm internal diameter and 5 µm particle size) was used. The mobile phase was ACN:H_2_O (80:20), and the flow rate was 1 mL/min at a temperature of 40 °C. The equipment was calibrated at wavelengths of 215 and 220 nm, and the analysis time was 30 min. The standards used were β-sitosterol, stigmasterol, α-amyrin, ursolic acid, oleanolic acid, carnosol, and 7-keto. To identify and quantify the two types of metabolites, the external standards (Sigma, Co., Mexico City, Mexico) mentioned above were used; solutions of pure compounds were prepared at concentrations of 0.08, 0.16, 0.32, 0.64, and 1.28 mg/mL in HPLC-grade methanol. Using the readings of each series of standard solutions for peak absorption areas and flavonoid or terpenoid concentration, linear regression equations were obtained to calculate the content of the compounds in the samples. Both methods were based on the approach of Aguiñiga-Sánchez et al. (2017), albeit with modifications [21].

### 2.5. Pharmacological Evaluations

#### 2.5.1. Animals

The pharmacological evaluation was carried out in male CD-1 mice. The mice were provided by the biotherium of the Faculty of Sciences. All experimental procedures were carried out in accordance with the approved Mexican guidelines, NOM-062-ZOO-1999, and International Standards for the Care and Use of Laboratory Animals in Research. The protocol was accepted by the Committee on Academic Ethics and Scientific Responsibility (CEARC) of the Faculty of Sciences, UNAM, under the folio PI_2021_08_02_Aguirre. Extracts and the reference drug were suspended in 0.9% s.s. and Tween 80. Each group of six animals was administered with vehicle (s.s.), the reference drug (DCF, 10 mg/kg), and EtOAc extracts (300 mg/kg). All treatments were administered orally (p. o.) in a volume of 10 mL/kg.

#### 2.5.2. Formaline Test

This test consisted of an intraplantar injection of 20 µL of 1% formalin into the right hindlimb of each mouse to produce a licking behaviour. Subsequently, the mouse’s licking of the injected paw with the nociceptive agent was recorded for 1 min, every 5 min, for a total period of 30 min. Two phases were recorded in this test: neurogenic (0–10 min) and inflammatory (10–30 min) phases. A significant decrease in either phase was interpreted as demonstrative of an antinociceptive effect.

### 2.6. Statistical Analysis 

Data for terpenoids and flavonoids concentrations are presented as the mean ± standard deviation (SD) of µg compound/mg extract. Statistical analysis was carried out using ANOVA, followed by Tukey’s post hoc test, to compare the concentrations of the different metabolites in each location. The data were then analysed using RStudio version 2023.03.0 (Boston, MA, USA) through principal components analysis (PCA) and correspondence canonical analysis (CCA) to select the most contrasting sites according to their secondary metabolite content. Data from pharmacological evaluations were presented as the mean ± standard error (SEM) of each treatment. Statistical analysis was via by ANOVA, followed by Dunnett’s post hoc test, to compare treatments against the vehicle group. A value of *p* > 0.05 was considered significant. The analysis was performed using the GraphPad version 8 program (GraphPad Software, La Jolla, CA, USA). 

## 3. Results

### 3.1. Quantification of Terpenoids and Flavonoids from S. semiatrata via HPLC-DAD

**Figure 2:**
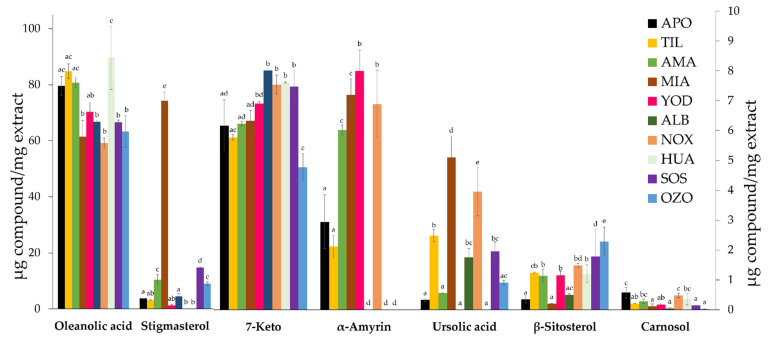
*S. semiatrata* terpenoid content from ten locations. The bars represent the mean ± standard deviation of three replicates. Oleanolic acid and stigmasterol contents are presented on the left *Y*-axis, and the other compounds are presented on the right *Y*-axis. For each location, values with different minuscule letters are significantly different, according to Tukey’s test *p* < 0.05.

Analysis showed the presence of the terpenoids oleanolic acid, stigmasterol, 7-keto, α-amyrin, ursolic acid, β-sitosterol, and carnosol, as well as the flavonoids quercetin, catechin, and naringenin (Supplementary Table S1). According to Tukey’s analysis, the content of the identified metabolites varied across the 10 locations (Figure 2). Oleanolic acid (89.60–59.20 µg/mg) and 7-keto (8.01–4.76 µg/mg) were found in high concentrations and had low variability across the ten locations. With respect to β-sitosterol (2.29–0.20 µg/mg) and carnosol (0.57–0.02 µg/mg), which were also present in all ten locations, they were in lower amounts and had higher variability. Ursolic acid, stigmasterol, and α-amyrin were only present in the extracts sourced from some collection localities. 

**Figure 3:**
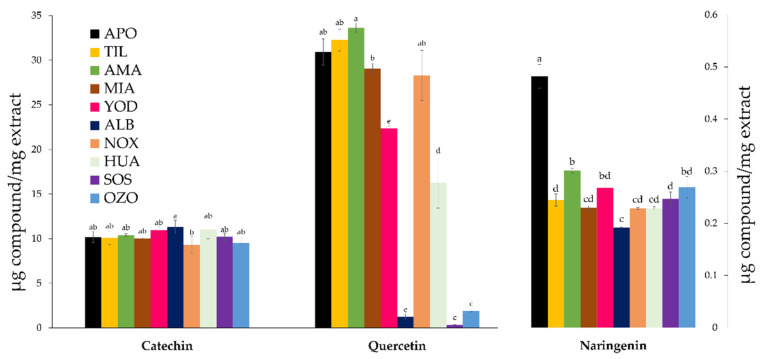
*S. semiatrata* flavonoids content from ten locations. The bars represent the mean ± standard deviation of three replicates. The content of catechin and quercetin is presented on the left *Y*-axis and that of the other compounds on the right *Y*-axis. For each location, values with different minuscule letters are significantly different according to Tukey’s test *p* < 0.05.

Oleanolic acid is the terpenoid found in the highest concentration in all extracts, with the highest concentration identified in the Santiago Huauclilla sample. As for 7-keto, its presence is notable in the San Lorenzo Albarradas sample (8.01 ± 0.08 µg/mg), where the dominant vegetation is Xeric scrub, and the Santiago Huauclilla sample (7.62 ± 0.02 µg/mg), where the plant collection was performed at a site with soil used for agriculture. In sum, the locality of Santiago Huauclilla stands out based on the presence of oleanolic acid and 7-keto, as well as the absence of stigmasterol, α-amyrin, and ursolic acid. Regarding flavonoids, the presence of catechin, quercetin, and naringenin was detected. According to Tukey’s analysis, the concentrations differed across the 10 locations (Figure 3). The presence of catechin (11.30–9.30 µg/mg) and naringenin (0.48–0.19 µg/mg) are the most stable variables, whereas quercetin is found in high concentrations in the Amatlán locality (34.81 ± 0.53 µg/mg) and low concentrations in localities such as San Lorenzo Albarradas (1.23 ± 0.32 µg/mg), San Juan Sosola (0.35 ± 0.00 µg/mg), and Santo Domingo Ozolotepec (1.90 ± 0.08 µg/mg).

### 3.2. Principal Component Analysis (PCA) of Secondary Metabolite Content

**Figure 4:**
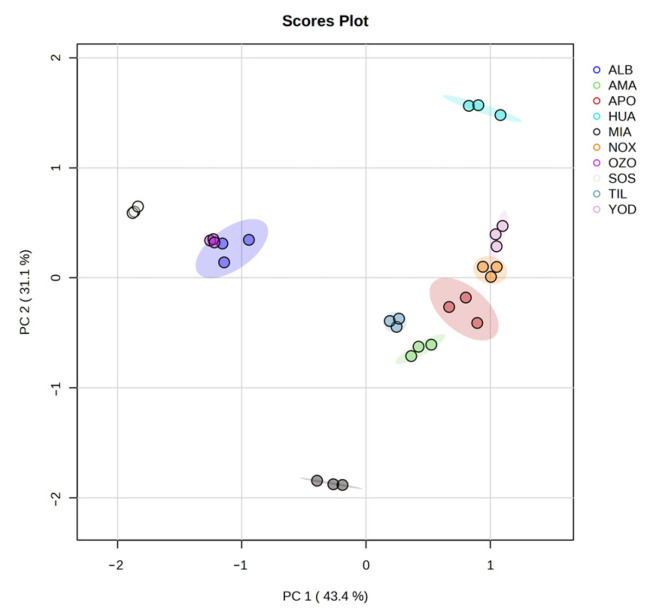
PCA scatter plot of the secondary metabolite content. The horizontal axis shows PC1, which mainly represents ursolic acid, quercetin, and α-amyrin, and it accounts for 43.4% of the variation. The vertical axis displays PC2, which mainly represents oleanolic acid, quercetin, and ursolic acid, and it accounts for 31.1% of the variation. This figure was created using Metabolanalyst 5.0.

In the PCA of 10 compounds, the first 2 principal components were selected. The contribution of the eigenvalues of these two components was 74.5%. A 2D diagram was used to visualize the characteristics of the identified secondary metabolite content. PC1 mainly represented the content of ursolic acid, quercetin, and α-amyrin, which accounted for 43.4% of the variation, while PC2 mainly represented the content of oleanolic acid, quercetin, and ursolic acid, which accounted for 31.3% of the variation. Similar characteristics were found for the contents identified in Magdalena Yodocono, Nochixtlán, Santiago Apoala, Amatlán, and Santiago Tilantongo. On the other hand, the localities of San Lorenzo Albarradas, Ozolotepec, and San Juan Sosola also showed similarities, while Miahuatlán and Santiago Huauclilla were the most different concentrations (Figure 4).

### 3.3. Canonical Correspondence Analysis (CCA) between Climatic Variables and Secondary Metabolite Content

**Figure 5:**
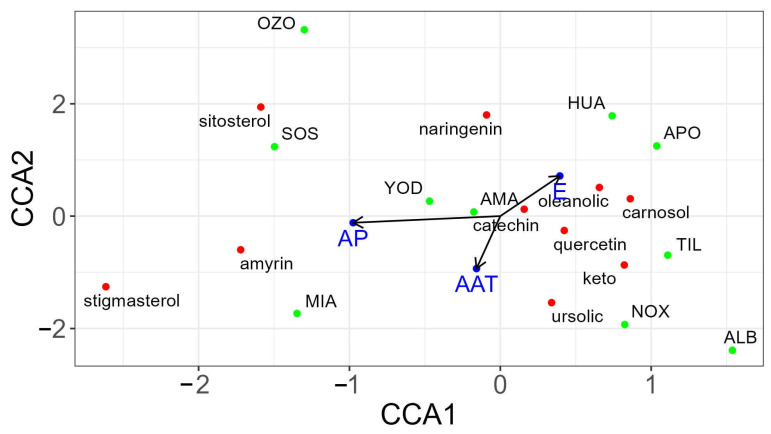
Canonical correspondence analysis between climatic variables and secondary metabolite content. The total variance value of the response matrix (secondary metabolite content) is 0.55, and the variance value explained based on the response matrix (environmental variables) is 0.16. The eigenvalues of axis 1 and axis 2 are 0.07 and 0.05, respectively.

The CCA reveals that precipitation and temperature variables are more synergistic with each other than with altitude, with which they have an antagonistic relationship (Figure 5). Metabolites such as stigmasterol and α-amyrin have a stronger correlation between precipitation, 7-keto, and ursolic acid and temperature, while naringenin and β-sitosterol have a strong correlation with altitude. It can be observed that HUA is mostly influenced by altitude, whereas MIA is mainly influenced by precipitation and temperature.

The values obtained after statistical analysis show that climatic variables, such as elevation, annual precipitation, and annual average temperature, in this case, do not have a significant explanatory power regarding the variation in the concentration of the quantified secondary metabolites.

### 3.4. Vegetation Type and Variation in Terpenoids and Flavonoids

**Figure 6:**
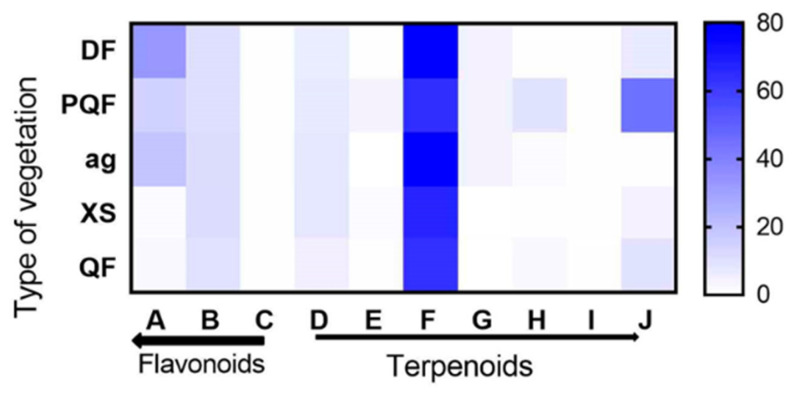
Heat map related to the concentrations of different terpenoids (D—7-keto, E—ursolic acid, F—oleanolic acid, G—α-amyrin, H—β-sitosterol, I—carnosol, J—stigmasterol) and flavonoids (A—quercetin, B—catechin, C—naringenin) in five different types of vegetation (DF—Deciduous Forest, QF—*Quercus* Forest, PQF—*Pinus*–*Quercus* Forest, Ag—Agriculture, XS—Xeric Scrub). Concentration levels (µg/mg) are indicated by the shift from blue to white, with dark blue indicating the highest values.

The distribution of terpenoid and flavonoid contents showed variation with respect to vegetation type in the different *S. semiatrata* collection sites. There were significant differences for all compounds in the different vegetation types. In the case of terpenoids, oleanolic acid showed the highest values regarding the different vegetation types, being highest in the deciduous forests at 80.18 µg/mg. Regarding flavonoids, quercetin showed the highest values under these conditions, except in the xeric scrub and *Quercus* Forest. The correlation between terpenoids and flavonoids across all locations and vegetation types (Figure 6, Supplementary Table S2) showed that a positive correlation existed between quercetin and naringenin (Pearson’s 0.42, *p*-value 0.0276) and carnosol (Pearson’s 0.50, *p*-value 0.0073), while a negative correlation existed with stigmasterol (Pearson’s −0.55, *p*-value 0.0031). Catechin was negatively correlated with carnosol (Pearson’s −0.44, *p*-value 0.0208), while oleanolic acid had a negative correlation with stigmasterol (Pearson’s −0.40, *p*-value 0.0379). Finally, α-amyrin had a negative correlation with β-sitosterol (Pearson’s −0.40, *p*-value 0.0405).

### 3.5. Evaluation of the Antinociceptive Effect

**Figure 7:**
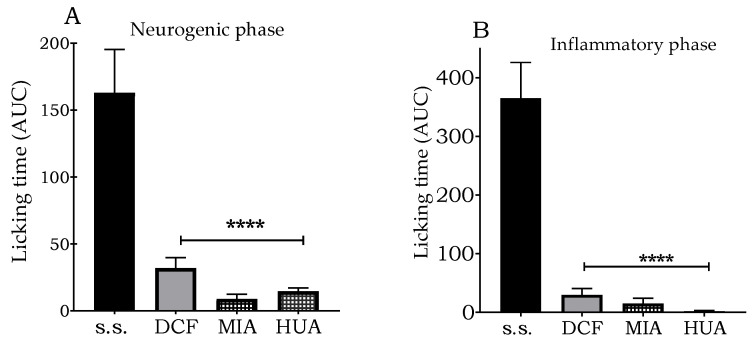
Antinociceptive effect of ethyl acetate extracts of *S. semiatrata*. Bars represent the mean ± standard error of six animals. Asterisks indicate significant differences with respect to vehicle, with ANADEVA performed in this analysis, followed by Dunnett’s test. Saline solution (s.s.), diclofenac (DCF), Miahuatlán (MIA), and Santiago Huauclilla (HUA). (**A**) Neurogenic phase; (**B**) Inflammatory phase **** *p* < 0.0001.

The evaluation of the antinociceptive effect was carried out by performing the formalin test on the extracts sourced from the localities of Miahuatlán and Santiago Huauclilla. Despite being the most contrasting localities in terms of secondary metabolite content, the extracts showed significant antinociceptive activity in both the inflammatory (Figure 7A) and neurogenic phases (Figure 7B) at a dose of 300 mg/kg, p. o.

## 4. Discussion

Plants produce many secondary metabolites for defence, communication, and competition with other plants. The production of these compounds is sufficiently important that around 200,000 have been isolated and identified [22]. Among the key metabolites for survival are those that are produced in response to stress caused by biotic and abiotic factors: within the first type are those that respond to defence against herbivores, bacteria, viruses, or other plants, while in the second type are those that are produced in response to temperature, ultraviolet radiation, drought, salinity, nutrient levels, etc. [22,23,24]. The type and concentration of metabolites depend on the species, organ, phenology, and environment in which they develop [20]. In this study, compounds from *S. semiatrata* collected in different localities were evaluated, and we analysed the influence of environmental variation on the in vivo anti-nociceptive effects of the extract. HPLC-DAD analysis showed higher content of terpenoids than flavonoids in all ten locations, which was expected, as terpenoids are the most diverse compounds found in plants [25]. The chemical constituents found in this species have similar activities for plants, such as pathogen defence, protection against oxidative stress caused by ultraviolet irradiation, repellence against herbivores, and growth regulation, although it has been observed that the responses to environmental changes in terms of compound increase or decrease may be different [26,27]. As for the terpenoids, oleanolic acid, 7-keto, β-sitosterol, and carnosol were identified in extracts sourced from all localities, with oleanolic acid standing out as the most abundant example, with concentrations ranging between 59.20 and 89.60 µg/mg, while 7-keto was present in similar concentrations in all locations. Of both terpenes, one has a wide occurrence within angiosperms and the other is more restricted in distribution [8,28]. In terms of terpenoid and flavonoid content, the MIA and HUA localities were showed greater contrast. In the former type, seven terpenoids and all three flavonoids were identified in its extracts. This result was probably due to the fact that the production of a greater number of secondary metabolites has been observed in plants that grow in temperate environments than in those that develop in dry environments, although in the latter case, the existing metabolites are more specific, and this result seemed to be corroborated when it was observed that the *S. semiatrata* localities with the greatest number and concentration of metabolites were those that thrived in the *Pinus*–*Quercus* Forest, which had the highest rainfall and average temperatures between 19 and 21 °C. With respect to HUA, the highest number of terpenoids was found, since stigmasterol, α-amyrin, and ursolic acid were not identified, while three flavonoids were found, which probably play an important role in the survival of this sage, which is distributed in soil used for agriculture and without tree or shrub vegetation, in which it competes with weeds. Consequently, irradiation was high and precipitation was low. This result is consistent with the results of the principal component analysis, which showed that the most contrasting locations in terms of secondary metabolite content are Miahuatlán and Santiago Huauclilla. Several studies have reported the great diversity of terpenoids and phenolic compounds in the different species of the genus *Salvia* [29,30]. Also, within the same species, variation in essential oils has been reported in wild specimens, such as *Salvia aramiensis* L., *S. chloroleuca* Rech f. and Aell, *S. officinalis* L., and *S. verbenaca* L. In general, in *S. aramiensis*, it was observed that the radii of concentrations varied little over the years: in *S. chloroleuca*, the population at lower altitudes contained up to twice the amount of β-pinene of populations at higher altitudes; for *S. officinalis*, temperature was positively correlated with the amount of sesquiterpenes and negatively correlated with the amount of bicyclic monoterpenes; and, finally, for S. verbenaca, the variation was attributed to differences in soil composition [31,32,33,34]. Regarding phenolic compounds, a 2019 study reported variations in the content of phenolic acids and flavonoids in *S. officinalis* sourced from different crops [35]. Likewise, studies of *S. miltiorrhiza* crops across different regions of China indicate that soil physicochemical properties and genotype affect the content of active components, such as rosmarinic acid, salvianolic acid B, and various tanshinones [36,37]. On the other hand, the trend in the CCA shows that in Santiago Huauclilla, the factor with the greatest impact on the composition of secondary metabolites is elevation, which is contrary to the trend in Miahuatlán, where annual precipitation and mean annual temperature have a greater influence, although no statistically significant explanation can be given for this result. According to the results obtained through the correlation of vegetation type and the concentration of terpenoids and flavonoids, a variation in the content of these compounds is observed. In localities with *Pinus*–*Quercus* Forest vegetation type, there was a higher presence and diversity of compounds because this environment offers favourable conditions for their synthesis and accumulation [38]. Furthermore, it was found that there is a positive correlation with the flavonoid group and a negative correlation with the terpenoid group. This result suggests that there may be synergies between substances, regardless of the type of locality and vegetation type. Finally, the metabolites identified and quantified in this work have previously been assessed in past individual studies, which corroborated their antinociceptive and anti-inflammatory effects in various pain tests (writhing, formalin test, hot plate test, tail immersion test, carrageenan-induced paw edema, among others); these metabolites include oleanolic and ursolic acids, 7-keto, and quercetin, amongst others [8,11,39]. The role of these compounds in antinociceptive and anti-inflammatory activity has been proven. Pentacyclic triterpenes, such as ursolic acid, oleanolic acid, and α-amyrin, inhibit the expression of nitric oxide synthase (iNOS), cyclo-oxygenase 2 (COX-2), tumour necrosis factor (TNF-α), histone deacetylase (HDAC), and nuclear factor kappa (NF-κβ) [40]. Regarding neo-clerodane diterpenes (important constituents of Mexican salvias), studies report their interaction with κ-opioid and 5-HT_1A_ serotonin receptors [41,42,43]. On the other hand, flavonoids have been extensively studied and shown to be involved in several inflammatory pathways, such as the reduction in C-reactive protein, serum amyloid A protein, and soluble E-secretin [44]. Therefore, even if there is variation in the amount and/or absence of any of the compounds in the extracts acquired from different locations, the effect is preserved due to their synergy [20]. In this regard, it is important to mention that extracts of different polarities of various *Salvia* species (*S. amarissima*, *S. purpurea*, *S. tiliifolia*, *S. involucrata*, *S. fulgens*, *S. melissodora*, among others) have had good antinociceptive effects in pain models, presenting within their composition metabolites such as ursolic acid, stigmasterol, α-amyrin, β-amyrin, oleanolic acid, and β-sitosterol, as well as the flavonoids apigenin, kaempferol, catechin, naringenin, phlorizin, quercetin, and rutin [6,7,9,45].

## 5. Conclusions

Since plant metabolism (primary and secondary) varies in response to biotic and abiotic variables, a major concern isthe way in which this process influences the production of secondary metabolites with biological activity useful to humans; therefore, characterising the effects of environmental changes on secondary metabolism is important. In the case of *S. semiatrata*, no controlled experiment was carried out to measure these factors; however, by measuring the anti-nociceptive effect of 10 samples collected in different environments, which had different limiting factors, it was indirectly observed that this effect is the same, which corroborates with the notions that the secondary metabolites found have similar activities for the plant and the production of some metabolites is sufficient for the survival of the plant in different environments, explaining why many medicinally used plants, while grown in different circumstances, can maintain their beneficial effects in terms of curing diseases. Thus, although the contents of the terpenoids oleanolic acid, stigmasterol, 7-keto, α-amyrin, ursolic acid, β-sitosterol, and carnosol, as well as the flavonoids catechin, quercetin, and naringenin, that were identified and quantified through HPLC-DAD vary across the ten collection sites of *S. semiatrata*, these variations do not influence the samples’ antinociceptive effects.

## Figures and Tables

**Table 1:** Climatic factors of the different locations. Data were obtained from the INEGI (National Institute of Statistics and Geography). Abbreviations: E—Elevation, AP—Annual Precipitation, AAT—Annual Average Temperature, DF—Deciduous Forest, QF—*Quercus* Forest, PQF—*Pinus-Quercus* Forest, Agr—Agriculture, XS—Xeric Scrub.

Location	Voucher Number FCME	Vegetation Types	E (masl)	AP (mm)	AAT (°C)
APO	181853	DF	1968	597.8	14.0
TIL	181854	QF	2220	708.6	18.5
AMA	181855	DF	1629	943.6	24.6
MIA	181856	PQF	1556	1002.6	19.8
YOD	181857	Agr	2303	1008.8	17.3
ALB	181858	XS	1749	570.4	23.3
NOX	181859	QF	2130	689.4	20.9
HUA	181860	Agr	2168	597.8	15.7
SOS	181861	PQF	1920	1104.8	18.6
OZO	181862	DF	2371	816.5	17.5

## Data Availability

Data regarding climatic factors from the different locations and types of vegetation were obtained from the National Institute of Statistics and Geography (INEGI) [Accessed on 11 January 2023: https://www.inegi.org.mx/temas/climatologia/#Mapa].

## Supplementary Materials

The supporting information can be downloaded via the following link: https://www.mdpi.com/article/10.3390/metabo13070866/s1, Table S1: Terpene and flavonoid concentrations in ethyl acetate extracts of *S. semiatrata* collected from 10 sites in Oaxaca. Abbreviations: NI—non-identified, Table S2: Pearson’s correlation matrix between flavonoid and terpene concentrations in ethyl acetate extract of *S. semiatrata*.

## References

[1] Ramamoorthy T.P., Elliott M., Ramamoorthy T.P., Bye R., Lot A., Fa J. (1998). Lamiaceae de México: Diversidad, distribución, endemismo y evolución. Diversidad biológica de México: Orígenes y Distribución.

[2] Martinez-Gordillo M., Fragoso-Martínez I., García-Peña M., Montiel O. (2013). Genera of Lamiaceae from Mexico, diversity and endemism. Rev. Mex. Biodivers..

[3] Argueta A., Cano L., Rodarte M. (1994). Atlas de las Plantas de la Medicina Tradicional Mexicana.

[4] Ortiz-Mendoza N., Aguirre-Hernández E., Fragoso-Martínez I., González-Trujano M.E., Basurto-Peña F.A., Martínez-Gordillo M.J. (2022). A Review on the Ethnopharmacology and Phytochemistry of the Neotropical Sages (*Salvia* Subgenus *Calosphace*; Lamiaceae) Emphasizing Mexican Species. Front. Pharmacol..

[5] Wu Y.B., Ni Z.Y., Shi Q.W., Dong M., Kiyota H., Gu Y.C., Cong B. (2012). Constituents from *Salvia* species and their biological activities. Chem. Rev..

[6] Moreno-Pérez G., González-Trujano M.E., Martínez-Gordillo M.J., Miguel-Chávez R., Basurto-Peña F.A., Dorazco González A., Aguirre-Hernández E. (2019). Amarisolide A and pedalitin as bioactive compounds in the antinociceptive effects of *Salvia circinata* (Lamiaceae). Bot. Sci..

[7] Cuevas-Morales C., Zavala-Ocampo L.M., San Miguel-Chávez R., González-Trujano M.E., Basurto-Peña F.A., Muñoz-Ocotero V., Aguirre-Hernández E. (2022). Pharmacological Evaluation of the Antinociceptive Activity and Phytochemical Analysis of Active Extracts of *Salvia purpurea* Cav. Bot. Sci..

[8] Ortiz-Mendoza N., Zavala-Ocampo L.M., Martínez-Gordillo M.J., González-Trujano M.E., Basurto-Peña F.A., Bazany-Rodríguez I.J., Rivera-Chávez J.A., Dorazco-González A., Aguirre-Hernández E. (2020). Antinociceptive and anxiolytic-like effects of a neo-clerodane diterpene from *Salvia semiatrata* aerial parts. Pharm. Biol..

[9] Anaya-Alvarado A. (2022). Evaluación de la Actividad Analgésica de *Salvia involucrata* y Análisis Químico del Extracto Active. Bachelor’s Thesis.

[10] Githinji C.G., Mbugua P.M., Kanui T.I., Kariuki D.K. (2012). Phytochemical and analgesic evaluation of *Mondia whytei* (hook.f) root. J. Pharmacogn. Phytother..

[11] Rodrigues M.R.A., Kanazawa L.K.S., Neves T.L.M.D., Silva C.F.D., Horst H., Pizzolatti M.G., Werner M.F.D.P. (2012). Antinociceptive and anti-inflammatory potential of extract and isolated compounds from the leaves of *Salvia officinalis* in mice. J. Ethnopharmacol..

[12] Hirota B.C.K., Paula C.D.S., De Oliveira V.B., Da Cunha J.M., Schreiber A.K., Ocampos F.M., Miguel M.D. (2016). Phytochemical and antinociceptive, anti-inflammatory, and antioxidant studies of *Smilax larvata* (Smilacaceae). Evid. -Based Complement. Altern. Med..

[13] Xu W., Zhou Q., Yao Y., Li X., Zhang J., Su G., Deng A. (2016). Inhibitory effect of gardenblue blueberry (*Vaccinium ashei* Reade) anthocyanin extracts on lipopolysaccharide-stimulated inflammatory response in RAW 264.7 cells. J. Zhejiang Univ. Sci. B.

[14] do Nascimento J.E.T., de Morais S.M., de Lisboa D.S., de Oliveira Sousa M., Santos S.A.A.R., Magalhães F.E.A., Campos A.R. (2018). The orofacial antinociceptive effect of kaempferol-3-O-rutinoside, isolated from the plant *Ouratea fieldingiana*, on adult zebrafish (*Danio rerio*). Biomed. Pharmacother..

[15] Forouzanfar F., Hosseinzadeh H. (2018). Medicinal herbs in the treatment of neuropathic pain: A review. Iran. J. Basic Med. Sci..

[16] Islam S., Shajib M.S., Rashid R.B. (2019). Antinociceptive activities of *Artocarpus lacucha* Buch-ham (Moraceae) and its isolated phenolic compound, catechin, in mice. BMC Complement. Altern. Med..

[17] Wang M., Ma C., Chen Y., Li X., Chen J. (2019). Cytotoxic Neo-clerodane diterpenoids from *Scutellaria barbata* D.D_ON_. Chem. Biodivers..

[18] González-Chávez M., Alonso-Castro A., Zapata-Morales J., Arana-Argáez V., Torres-Romero J., Medina-Rivera Y., Sánchez-Mendoza E., Pérez-Gutiérrez S. (2018). Anti-inflammatory and antinociceptive effects of tilifodiolide, isolated from *Salvia tiliifolia* Vahl (Lamiaceae). Drug Dev. Res..

[19] Javed F., Jabeen Q., Aslam N., Awan A.M. (2020). Pharmacological evaluation of analgesic, anti-inflammatory and antipyretic activities of ethanolic extract of *Indigofera argentea* Burm. f. J. Ethnopharmacol..

[20] Wink M., Wink M. (2010). Introduction: Biochemistry, Physiology and Ecological Functions of Secondary Metabolites. Annual Plant Reviews Biochemistry of Plant Secondary Metabolism.

[21] Aguiñiga I., Cadena-Iñiguez J., Santiago-Osorio E., Gómez-García G., Mendoza-Núñez V.M., Rosado-Pérez J., Ruíz-Ramos M., Cisneros V., Ledesma-Martínez E., Delgado-Bordonave A. (2017). Chemical analyses and in vitro and in vivo toxicity of fruit methanol extract of *Sechium edule* var. *nigrum spinosum*. Pharm. Biol..

[22] Carrera F.P., Noceda C., Maridueña-Zavala M.G., Cevallos-Cevallos J.M. (2021). Metabolomomics, a powerful tool for understanding Plant abiotic stress. Agronomy.

[23] Nakabayashi R., Yonekura-Sakakibara K., Urano K., Suzuki M., Yamada Y., Nishizawa T., Matsuda F., Kojima M., Sakakibara H., Shinozaki K. (2014). Enhancement of oxidative and drought tolerance in *Arabidopsis* by overaccumulation of antioxidant flavonoids. Plant J..

[24] Arbona V., Manzi M., de Ollas C., Gómez-Cadenas A. (2013). Metabolomics as a tool to investigate abiotic stress tolerance in plants. Int. J. Mol. Sci..

[25] Sülsen V.P., Lizarraga E., Mamadalieva N.Z., Lago J.H.G. (2017). Potential of Terpenoids and Flavonoids from Asteraceae as Anti-Inflammatory, Antitumor, and Antiparasitic Agents. Evid. -Based Complement. Altern. Med..

[26] Bano A., Qadri T.A., Mahnoor, Khan N. (2023). Bioactive metabolites of plants and microbes and their role in agricultural sustainability and mitigation of plant stress. S. Afr. J. Bot..

[27] Anjali, Kumar S., Korra T., Thakur R., Arutselvan R., Kashyap A.S., Nehela Y., Chaplygin V., Minkina T., Keswani C. (2023). Role of plant secondary metabolites in defence and transcriptional regulation in response to biotic stress. Plant Stress.

[28] Kalaycıoğlu Z., Uzaşçı S., Dirmenci T., Erim F.B. (2018). α-Glucosidase enzyme inhibitory effects and ursolic and oleanolic acid contents of fourteen Anatolian *Salvia* species. J. Pharm. Biomed. Anal..

[29] Jash S., Gorai D., Roy R. (2016). *Salvia* genus and triterpenoids. Int. J. Pharm. Sci. Res..

[30] Lu Y., Foo Y. (2002). Polyphenolics of *Salvia*—A review. Phytochemistry.

[31] Türkmen M., Kaya D.A., Ayanoğlu F. Variations in essential oil main components of native grown *Salvia aramiensis* Rech fil. genotypes depending on years. Proceedings of the ICAMS Proceedings of the International Conference on Advanced Materials and Systems.

[32] Talebi S.M., Behzadpour A., Matsyura A. (2019). Morphological and essential oil variations among Iranian populations of *Salvia chloroleuca* (Lamiaceae). Biosyst. Divers..

[33] Myrtaj B., Dervishi A., Nuro A., Salihila J., Peci D. (2022). Climate influence and essential oils composition of *Salvia officinalis* in populations of southern Albania. Agric. For..

[34] Ben Farhat M., Sotomayor J.A., Jordán M.J. (2019). *Salvia verbenaca* L. essential oil: Variation of yield and composition according to collection site and phenophase. Biochem. Syst. Ecol..

[35] Duletić-Laušević S., Aradski A.A., Živković J., Gligorijević N., Šavikin K., Radulović S., Marin P.D. (2019). Evaluation of bioactivities and phenolic composition of extracts of *Salvia officinalis* L. (Lamiaceae) collected in montenegro. Bot. Serbica.

[36] Zhang C., Yang D., Liang Z., Liu J., Yan K., Zhu Y., Yang S. (2019). Climatic factors control the geospatial distribution of active ingredients in *Salvia miltiorrhiza* Bunge in China. Sci. Rep..

[37] He C., Han T., Liu C., Sun P., Liao D., Li X. (2023). Deciphering the effects of genotype and climatic factors on the performance, active ingredients and rhizosphere soil properties of *Salvia miltiorrhiza*. Front. Plant Sci..

[38] Holopainen J.K., Virjamo V., Ghimire R.P., Blande J.D., Julkunen-Tiitto R., Kivimäenpää M. (2018). Climate Change Effects on Secondary Compounds of Forest Trees in the Northern Hemisphere. Front. Plant Sci..

[39] Zarenezhad E., Abdulabbas H.T., Kareem A.S., Kouhpayeh S.A., Barbaresi S., Najafipour S., Mazarzaei A., Sotoudeh M., Ghasemian A. (2023). Protective role of flavonoids quercetin and silymarin in the viral-associated inflammatory bowel disease: An updated review. Arch. Microbiol..

[40] de-Almeida S.C.X., da-Silva A.C.F., Sousa N.R.T., Amorim I.H.F., Leite B.G., Neves K.T.R., Costa J.G.M., Felipe C.F.B., de-Barros-Viana G.S. (2019). Antinociceptive and anti-inflammatory activities of a triterpene-rich fraction from *Himatanthus drasticus*. Braz. J. Med. Biol..

[41] Moreno-Pérez G.F., González-Trujano M.E., Hernández-León A., Valle-Dorado M.G., Valdés-Cruz A., Alvarado-Vásquez N., Aguirre-Hernández E., Salgado-Ceballos H., Pellicer F. (2022). Antihyperalgesic and Antiallodynic Effects of Amarisolide A and *Salvia amarissima* Ortega in Experimental Fibromyalgia-Type Pain. Metabolites.

[42] Rodríguez-Hahn L., Esquivel B., Cárdenas J., Arnason J.T., Mata R., Romeo J.T. (1995). Neo-Clerodane Diterpenoids from American *Salvia* Species. Phytochemistry of Medicinal Plants. Recent Advances in Phytochemistry.

[43] Lovell K., Prevatt-Smith K., Lozama A., Prisinzano E., Nagase H. (2011). Synthesis of Neoclerodane diterpenes and their pharmacological effects. Chemistry of Opioids.

[44] Safe S., Jayaraman A., Chapkin R.S., Howard M., Mohankumar K., Shrestha R. (2021). Flavonoids: Structure-function and mechanisms of action and opportunities for drug development. Toxicol. Res..

[45] Liu J. (1995). Pharmacology of olenolic acid and ursolic acid. J. Ethnopharmacol..

