# Supplementary material for: Variation in Terpenoid and Flavonoid Content in Different Samples of Salvia semiatrata Collected from Oaxaca, Mexico, and Its Effects on Antinociceptive Activity
Source: Metabolites. 2023 Jul 20;13(7):866. doi: 10.3390/metabo13070866 (PMC10384789; doi:10.3390/metabo13070866)
Supplement: Supplementary file 1 [file metabolites-13-00866-s001.zip › metabolites-2509878-supplementary.pdf]

Table S1. Terpene and flavonoid concentrations in ethyl acetate extracts of *S. semiatrata* collected from 10 sites in Oaxaca. Abbreviations: n.i.-non- identified.

	Terpenoids							Flavonoids		
	(µg compound/mg extract ± S.D.)									
Location	Oleanolic Acid	Stigmasterol	7-Keto	α-amyrin	Ursolic Acid	β-sitosterol	Carnosol	Quercetin	Catechin	Naringenin
APO	79.60±0.33	3.80±0.02	6.16±0.86	2.94±0.90	0.33±0.05	0.35±0.12	0.57±0.18	30.92 ± 1.48	10.19 ± 0.63	0.48 ± 0.22
TIL	84.84±2.63	3.14±0.15	5.77±0.09	2.11±0.37	2.48±0.21	1.24± 0.20	0.21±0.00	32.26±1.23	10.09±0.74	0.24± 0.11
AMA	80.76±1.73	10.46±1.82	6.22±0.07	6.02±0.17	0.56±0.02	1.14±0.21	0.28±0.07	34.81± 0.53	10.41± 0.13	0.30±0.00
MIA	61.47±5.72	74.26±3.12	6.33±0.35	7.19±0.53	5.10±0.72	0.20±0.01	0.11±0.07	29.04±0.58	10.02± 0.08	0.23± 0.00
YOD	70.32±3.08	1.27±0.30	6.91±0.07	8.00±0.70	n.i.	1.16±0.13	0.18±0.01	22.35±0.31	10.98±0.40	0.27±0.01
ALB	66.74±0.40	4.52±0.90	8.01±0.08	n.i.	1.75±0.29	0.50±0.04	0.05±0.20	1.23±0.32	11.30±0.78	0.19± 0.00
NOX	59.20±1.80	n.i.	7.54±0.32	6.88±1.13	3.95±0.80	1.49±0.06	0.48±0.07	28.31±2.80	9.30± 0.99	0.23±0.00
HUA	89.60±11.21	n.i.	7.62±0.02	n.i.	n.i.	1.20±0.30	0.36±0.19	16.28±2.86	11.02±1.01	0.23± 0.00
SOS	66.62±0.80	14.81±0.35	7.47±0.56	n.i.	1.95±0.32	1.78±0.89	0.14±0.01	0.35±0.0	10.21±0.44	0.25±0.00
OZO	63.32±5.70	9.04±0.65	4.76±0.45	n.i.	0.91±0.07	2.29± 0.48	0.02±0.00	1.90±0.08	9.52±0.71	

Table S2. Pearson correlation matrix between flavonoid and terpene concentrations in ethyl acetate extract of *S. semiatrata*.

Variable(1)	Variable(2)	Pearson	p-valor
Quercetin	Catechin	-0.36	0.0651
Quercetin	Naringenin	0.42	**0.0276**
Quercetin	7-keto	-0.39	**0.0463**
Quercetin	Ursolic Acid	-0.35	0.0769
Quercetin	Oleanolic acid	0.35	0.0712
Quercetin	α-amyrin	0.07	0.7322

Quercetin	β-sitosterol	0.25	0.2023
Quercetin	Carnosol	0.50	**0.0073**
Quercetin	Stigmasterol	-0.55	**0.0031**
Catechin	Naringenin	-0.12	0.5372
Catechin	7-keto	0.14	0.4737
Catechin	Ursolic Acid	-0.38	0.0478
Catechin	Oleanolic acid	0.17	0.3856
Catechin	α-amyrin	-0.22	0.2741
Catechin	β-Sitosterol	-0.18	0.3817
Catechin	Carnosol	-0.44	**0.0208**
Catechin	Stigmasterol	-0.10	0.6296
Naringenin	7-keto	-0.51	**0.0069**
Naringenin	Ursolic Acid	-0.36	0.0628
Naringenin	Oleanolic acid	0.28	0.1635
Naringenin	α-amyrin	0.13	0.5041
Naringenin	β-Sitosterol	-0.20	0.3225
Naringenin	Carnosol	0.60	0.0009
Naringenin	Stigmasterol	-0.09	0.6474
7-keto	Ursolic Acid	-0.05	0.8043
7-keto	Oleanolic acid	-0.30	0.1311
7-keto	α-amyrin	-0.36	0.0643
7-keto	β-Sitosterol	0.26	0.1966
7-keto	Carnosol	-0.18	0.3640
7-keto	Stigmasterol	-0.26	0.1957
Ursolic Acid	Oleanolic acid	-0.60	**0.0010**
Ursolic Acid	α-amyrin	0.28	0.1577

Ursolic Acid	β-Sitosterol	0.02	0.9356
Ursolic Acid	Carnosol	-0.19	0.3408
Ursolic Acid	Stigmasterol	0.65	**0.0002**
Oleanolic acid	α-amyrin	-0.36	0.0644
Oleanolic acid	β-Sitosterol	-0.20	0.3165
Oleanolic acid	Carnosol	0.26	0.1862
Oleanolic acid	Stigmasterol	-0.40	**0.0379**
α-amyrin	β-Sitosterol	-0.40	**0.0405**
α-amyrin	Carnosol	0.13	0.5171
α-amyrin	Stigmasterol	0.33	0.0960
β-Sitosterol	Carnosol	-0.22	0.2683
β-Sitosterol	Stigmasterol	-0.01	0.9525
Carnosol	Stigmasterol	-0.35	0.0703
